# Dilemmas in the diagnosis and management of osteoporosis in a patient with alkaptonuria: Successful treatment with teriparatide

**DOI:** 10.1002/ccr3.6729

**Published:** 2022-12-27

**Authors:** Ismail C. Ebrahim, Thanh D. Hoang, Nicole O. Vietor, John P. Schacht, Mohamed K. M. Shakir

**Affiliations:** ^1^ Division of Endocrinology, Department of Medicine Walter Reed National Military Medical Center Bethesda Maryland USA; ^2^ Division of Endocrinology, Department of Medicine Uniformed Service University of the Health Sciences Bethesda Maryland USA; ^3^ Department of Genetics Walter Reed National Military Medical Center Bethesda Maryland USA

**Keywords:** alkaptonuria, fractures, osteoporosis, teriparatide

## Abstract

Management of osteoporosis in patients with alkaptonuria can be challenging. This is the first case report confirming the effectiveness of teriparatide following zoledronic acid therapy in treating osteoporosis and preventing fragility fractures in a patient with alkaptonuria.

## Graphical abstract

We report the first case of long‐term follow‐up of a 69‐year‐old woman who developed fragility fractures despite receiving bisphosphonate therapy and was successfully treated with teriparatide, an anabolic agent.

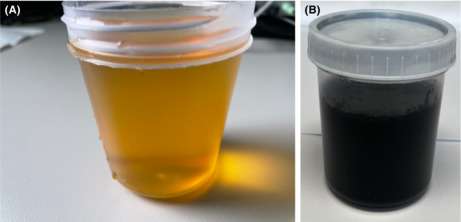

## 1 INTRODUCTION

It has been recognized that osteoporosis‐related fractures are common in patients with alkaptonuria (AKU). Therapy for osteoporosis in AKU is challenging since patients with AKU are susceptible to fractures despite bisphosphonate therapy.^1^, ^2^, ^3^, ^4^, ^5^ Ranganath et al recently reported successful treatment of osteoporosis with teriparatide in 2 patients with AKU.^6^ We report the first case of long‐term follow‐up of a 69‐year‐old woman who developed fragility fractures despite receiving bisphosphonate therapy and was successfully treated with teriparatide.

## 2 CASE PRESENTATION

**FIGURE 1:**
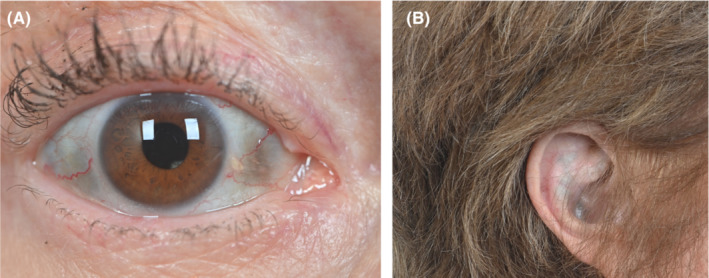
(A, B): Bluish black pigmentation of sclera (A) and ear (B)

**FIGURE 2:**
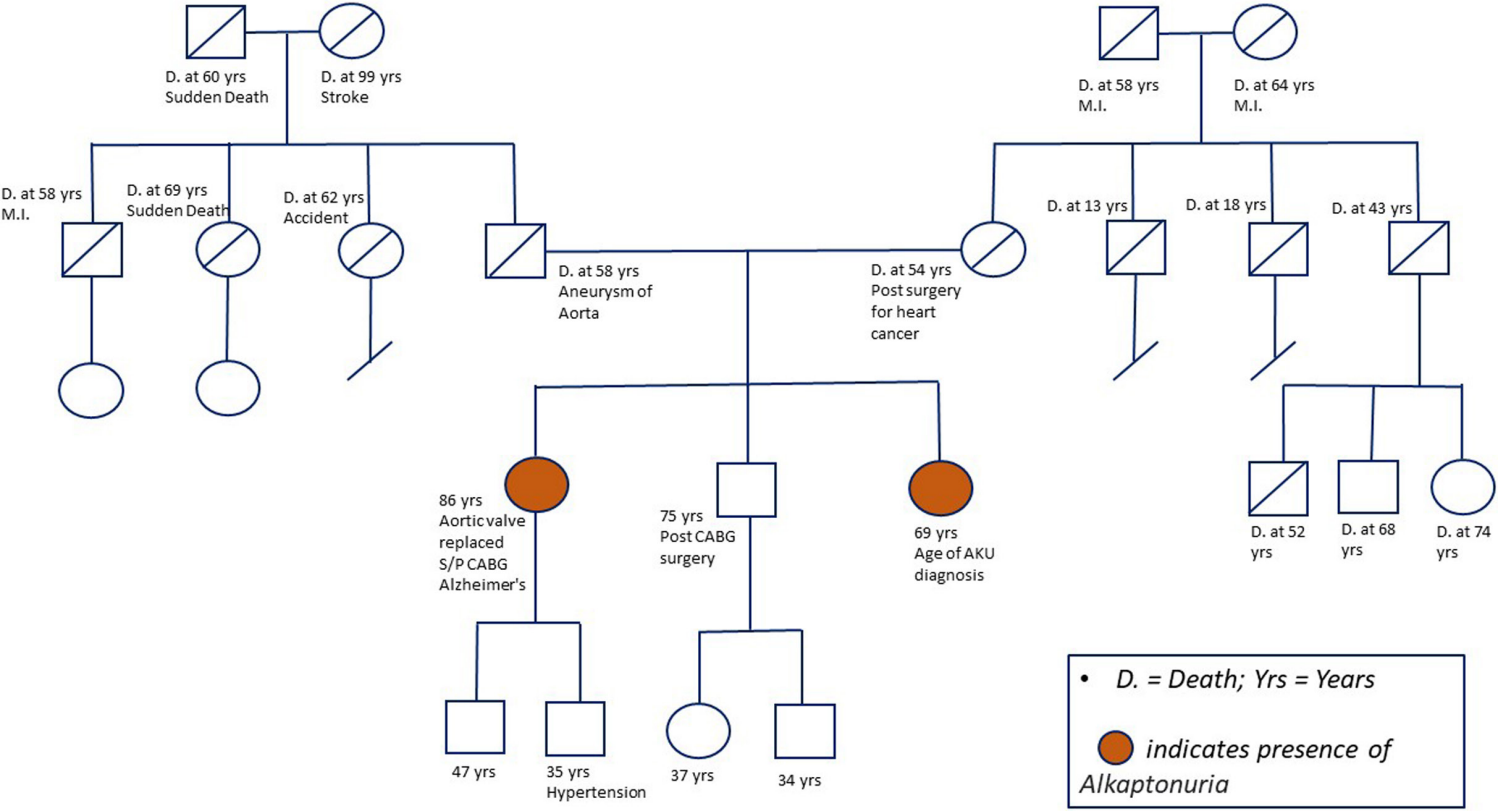
Family tree (pedigree)

A 69‐year‐old female with a history of AKU was evaluated for osteoporosis. She was diagnosed with AKU at the age of 29 years when the dermatologist noted bluish conjunctival hyperpigmentation (Figure 1A,B and Table 1). The patient experienced joint pains involving several joints and subsequently underwent joint replacements involving both knees, bilateral total hips, and bilateral shoulders. Past history included low back pain managed with celecoxib and tramadol. Family history (Figure 2) revealed her 85‐year‐old sister was diagnosed with AKU, had aortic valve replacement surgery, and underwent multiple joint replacement surgeries. On examination of the head/ears/eyes/nose/throat (HEENT): bluish black pigmentation of bilateral sclera and ears (Figure 1A,B). Heart: normal S1 (first heart sound) and S2 (second heart sound), late peaking systolic ejection murmur heard on right parasternal area.

**TABLE 1:** Showing baseline laboratory values

Serum		
Calcium (total)	9.4	8.4–10.2 mg/dl
Calcium (ionized)	5.1	4.8–5.6 mg/dl
Phosphorus	3.1	2.5–4.5 mg/dl
Magnesium	2.1	1.6–2.3 mg/dl
BUN	16	7–17 mg/dl
Creatinine	0.7	0.5–0.9 mg/dl
Alkaline phosphatase (total)	70	38–126 U/L
Alkaline phosphatase (bone specific)	11.2	8–32 ng/ml
Osteocalcin	11.2	8–32 ng/ml
Intact parathyroid hormone	40.7	11–65 pg/ml
C‐Telopeptide	397	104–1008 pg/ml
25‐Hydroxyvitamin D	42.6	30–100 ng/ml
1,25‐Dihydroxyvitamin D	38.9	21–65 pg/ml
Serum tyrosine	98.7	31.1–118.1 mcmol/L

Urine		
24 h calcium	198	100–250 mg
Urine organic acids 4‐OH Phenylacetic acid 4‐OH Phenylpyruvic acid	993	0–4 mmol/mol creatinine
	689	0–2 mmol/mol creatinine

*Note*: The routine laboratory tests were done either at the Walter Reed National Military Medical Center in Bethesda, MD, or at LabCorp™ in Burlington, NC. The quantitative plasma amino acids and urine organic acids were determined by LabCorp™, Burlington, NC.

**FIGURE 3:**
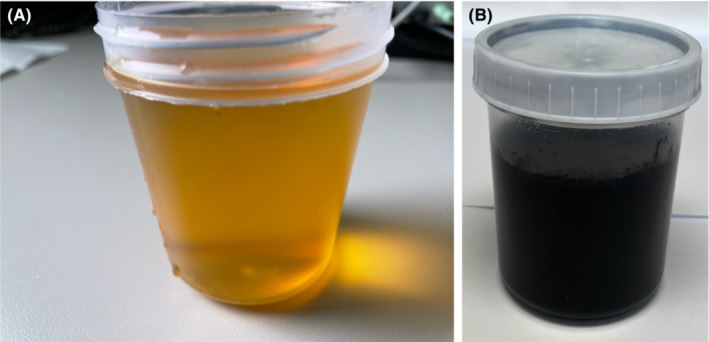
(A, B): Light yellow urine sample at time 0 (A); black urine after standing for 24 h (B)

Laboratory: (done at the age of 69 years) showed urine color changing to black after sunlight exposure (Figure 3A,B), normal 25‐hydroxyvitamin D, parathyroid hormone (PTH) levels, plasma amino acid analysis (patient on nitisinone): tyrosine 774.4 μmol/L (ref 27.8–83.3), osteocalcin 11.6 ng/ml, collagen cross‐linked C‐telopeptide 142 pg/ml, 24‐h urine organic acid markedly elevated (on nitisinone) although the levels were much lower compared to base line values (Table 1). A renal stone panel (Table 2) revealed mild hypercalciuria 4.1 mg/kg (ref <3.5 mg/kg), increased calcium oxalate, and calcium urate saturation. However, she was not treated with hydrochlorothiazide (Table 2).

**TABLE 2:** Showing kidney stone panel

Kidney stone, urine/saturation			Units	Ref range
Volume	24 HR URINE	1400	ml/24 h	600–1600
Calcium	24 HR URINE	261.8	mg/24 h	0.0–320.0
Sodium	24 HR URINE	112	mmol/24 h	39–258
Phosphate	24 HR URINE	604.8	mg/24 h	261.0–1078.0
Urate	24 HR URINE	504	mg/24 h	142–713
Potassium	24 HR URINE	48.3	mmol/24 h	14.0–95.0
Chloride	24 HR URINE	101	mmol/24 h	38–210
Citrate	24 HR URINE	930	mg/24 h	320–1240
Oxalate	24 HR URINE	32 (H)	mg/24 h	4–31
Magnesium	24 HR URINE	101	mg/24 h	12–293
Sulfate	24 HR URINE	18	mEq/24 h	0–30
Cystine	24 HR URINE	15.67	mg/24 h	2.10–58.00
Osmolality	24 HR URINE	456	mOsm/kg	300–900
Creatinine [mass/time] in 24 h urine	24 HR URINE	859.6	mg/24 h	800.0–1800.0
pH	24 HR URINE	5.6		4.5–8.0
Ammonia	24 HR URINE	31	mEq/24 h	Not Estab.
Calcium oxalate	24 HR URINE	9.71 (H)	Ratio	0.00–6.00
Calcium hydrogen phosphate dihydrate (brushite)	24 HR URINE	0.78	Ratio	0.00–3.00
Sodium urate saturation	24 HR URINE	2.36	Ratio	0.00–4.00
Sodium urate saturation	24 HR URINE	2.91 (H)	Ratio	0.00–1.20
Magnesium ammonium phosphate hexahydrate (struvite)	24 HR URINE	0.01	Ratio	0.00–1.00

An HGD (homogentisate 1,2‐dioxygenase) comprehensive gene analysis done further confirmed a positive HGD c.469 + 2T>C (p.7) (heterozygous) mutation further supporting the diagnosis of alkaptonuria.

Hospital course of osteoporosis (see Tables 3 and 4):

**TABLE 3:** History of osteoporosis treatment

Age treatment received	Medications	Comments
56–61 years	Oral alendronate 70 mg PO once a week for 2 years followed by ibandronate 150 mg PO once a month for the next 3 years.	Patient developed fragility fractures of ribs and a Colle's fracture of the left forearm at the age of 59 years, i.e., 3 years after treatment.
62–64 years	Teriparatide 20 mcg SC daily for the next 2 years	Patient tolerated the teriparatide well and had no fragility fractures.
65–69 years	Zoledronic acid 5 mg once a year for 5 years	Patient tolerated the treatment well and had no fragility fractures

**TABLE 4:** Results of DXA scan

Age (yrs)	L‐Spine			Total hip			Femoral neck			Forearm (distal 1/3rd radius)		
	BMD	T‐S	Z‐S	BMD	T‐S	Z‐S	BMD	T‐S	Z‐S	BMD	T‐S	Z‐S
56	1.361	3.0	4.8	0.706	−1.9	−0.6	0.577	−2.4	−0.9		N.D	
61	1.200	1.4	2.9	0.767	−1.5	−0.6	0.540	−2.9	−1.6	0.621	−1.2	0.2
63	1.365	2.9	4.5	0.534	−2.8	−1.4	0.673	−2.2	−1.1	0.567	−2.1	−0.7
64	1.361	2.9	4.5	0.566	−2.6	−1.1	0.703	−2.0	−0.8	0.554	−2.3	0.8
65	1.396	3.2	4.9	0.560	−2.6	−1.1	0.673	−2.2	−1.0		N.D	
68	1.455	3.4	5.3	0.769	−1.4	−0.2	0.557	−2.6	−1.6		N.D	
69	1.274	2.7	4.5		Bilateral hip replacement N.D					0.559	−2.2	0.2

*Note*: From the age of 56 years to 63 years, DXA scan was done using Hologic and 63 years to 69 years using hologic—.

Abbreviations: BMD, Bone Mineral Density; DXA, Dual Dual‐Energy X‐ray absorptiometry; T‐S, T‐scores; yrs, years; Z‐S, Z‐scores ND not done.

The patient was diagnosed with osteoporosis at the age of 56 years; Table 1 shows the baseline laboratory values prior to starting treatment for osteoporosis, Table 3 shows the history of osteoporosis treatment, and Table 4 shows the serial bone mineral density (BMD) values during the treatment with bisphosphonate therapy. Drug compliance for bisphosphonate therapy was verified by pill counting and by the response of bone resorption markers. Despite 5 years of compliance with bisphosphonate and nitisinone therapies, she developed fragility fractures at multiple sites with minimal trauma. Following these fractures, at the age of 61 years, she was treated with teriparatide 20 mcg daily subcutaneously for the next 2 years and then received annual zoledronic acid infusions for the subsequent 5 years. Serum calcium levels were monitored closely, and the patient did not develop hypercalcemia during teriparatide treatment.

Since the initiation of teriparatide therapy, she had no further fractures for following 9 years despite a physically active life. She also continued nitisinone 2 mg orally daily since the age of 56 years and had no side effects including keratopathy.

## 3 DISCUSSION

In AKU, mutations of a gene coding for homogentisate 1,2‐dioxygenase (HGD) lead to disruption of tyrosine metabolism and result in accumulation of homogentisic acid (HGA) which results in a multisystemic disorder. The patient reported here had several of the manifestations of AKU including darkening of urine upon standing, bluish discoloration of the eyes and ears, and degenerative joint diseases involving multiple joints and the spine.^1^, ^2^, ^3^, ^4^, ^5^, ^7^, ^8^, ^9^, ^10^, ^11^ It is reported that by age 64 years, 50% of individuals with alkaptonuria may develop nephrolithiasis,^9^ and the renal stone analysis usually shows calcium oxalate, phosphate, and carbonate.^7^, ^9^ However, computed tomography (CT) of the kidneys did not reveal any evidence of nephrolithiasis in our patient. A renal stone panel in our patient showed high normal urine citrate level although calcium oxalate and calcium urate saturations were elevated. These renal stone laboratory values are consistent with the previously reported calcium oxalate stones,^9^ and this is the first case report showing the urinary renal stone panel in a patient with alkaptonuria. The exact mechanism by which HGA deposition causes arthropathy is not clearly understood although it is suggested that the oxidized HGA deposits in the deeper layers of the articular cartilage may be a contributing factor. Additionally, the free radicals generated during the process of HGA oxidation may precipitate the inflammatory and degenerative processes, ultimately leading to tissue damage.^1^, ^7^, ^11^ Lysyl hydroxylase, an enzyme located in the cartilage tissue, is inhibited by the HGA oxidation process, and this may be a contributing factor.^11^ It is also possible that the bone matrix becomes more susceptible to pigmentation in response to tissue injury.^11^

Several variants of the HGD gene have been reported in AKU. An HGD comprehensive gene analysis done in our patient confirmed compound heterozygous pathogenic variants, HGD c.469 + 2T>C (p.7) and HGD c.1102A>G (p.Met368Val), consistent with a diagnosis of AKU. Ascher et al^12^ identified 28 novel variants of the HGD gene in 172 AKU patients, and a genotype–phenotype correlation study was performed for the three most frequent HGD variants. These investigators concluded that there was no difference in clinical symptoms, serum levels, or absolute urinary excretion of HGA.^12^

Despite the lack of HGA deposition in mineralized bone matrix without tissue injury, the risk of osteoporosis in AKU patients is high. Immobility of the spine due to pain hardened calcified ochronotic intervertebral discs and ochronosis in the adjacent articular cartilage of the vertebrae may occur. However, it is interesting to note that vertebral fractures in these patients are rather rare.^13^ Focal osteoporosis often leads to thinning of trabeculae and subchondral plates. Ranganath et al^4^ studied 15 patients with AKU and reported a nonvertebral fracture incidence of 53.3%. Our patient had several fractures involving multiple sites even while receiving oral bisphosphonate treatment. The overestimated BMD at the lumbar spine is likely related to intervertebral disc calcification and osteophyte formation,^3^ which we observed in our patient. Patients with ochronosis are more prone to fractures due to decreased BMD as seen in our patient who had ochronosis.^7^, ^14^, ^15^, ^16^, ^17^, ^18^, ^19^ Recent studies by Ranganath et al^18^, ^19^ showed nitisinone decreases HGA in AKU and reduces the rate of progression of AKU, mainly seen as combined ear and ocular bluish color progression. However, these investigators did not report on the effect of nitisinone on the skeletal manifestations. Since we did not measure the progression of ocular and ear ochronosis serially, it is difficult to assess the effect of this drug on ochronosis of the eye and ears. Additionally, nitisinone apparently had no effect on the progression of the skeletal manifestations of AKU in our patient. Our patient received nitisinone at the age of 56 years, and it is possible that treatment at an earlier age will be more effective.

Ranganath et al.^6^ reported that DXA (dual‐energy X‐ray absorptiometry) scan is not reliable in AKU patients due to extensive calcification of intervertebral discs as well as the extensive degenerative arthritis or joint replacements. In our patient, although an initial and serial DXA scans showed normal BMD at the lumbar spine, the BMD was likely falsely elevated; however, her hip BMD done at the age of 61 years showed osteoporosis (femoral neck T‐score −2.9). Follow‐up DXA scans could not be done at this site due to the joint replacement. Thus, the dilemmas involved in diagnosing osteoporosis seen in our patient with AKU are similar to those skeletal disorders such as diffuse interosseous skeletal hyperostosis (DISH) and ankylosing spondylitis. Alternative sites such as forearm BMD may have to be utilized. Alternatively, CT lumbar spine BMD measurements may be used.^9^ More recently, trabecular bone scan (TBS) has been utilized to measure BMD, although the diagnostic sensitivity of TBS in AKU remains to be established.

Management of osteoporosis in AKU patients thus presents several challenges to the clinician.^4^, ^18^, ^19^ Despite the appropriate use of bisphosphonate therapy initially, our patient developed multiple fragility fractures, confirming the failure to bisphosphonate therapy. Aliberti et al^4^ also concluded that there is no clinically significant difference in BMD after 2 years of bisphosphonate treatment. Similarly, nitisinone treatment does not improve osteoporosis or prevent fragility fractures associated with osteoporosis in these patients.^4^ After 5 years of treatment with bisphosphonates, our patient was treated with teriparatide. More recently, Ranganath et al in 2021 have also demonstrated the effectiveness of teriparatide in 2 AKU patients.^6^ Thus, our patient represents the third AKU patient with bisphosphonate‐resistant osteoporosis who was treated successfully with teriparatide. The anabolic actions of PTH occur because of its direct effects on cells of the osteoblast lineage and indirect effects through the induction of IGF‐I (insulin‐like growth factor‐1) and the suppression of sclerostin with the consequent enhancement of Wnt signaling and by suppressing Notch signaling.^20^

In conclusion, we recommend that osteoporosis in AKU be initially treated with teriparatide and later by intravenous zoledronic acid. Further studies involving larger number of patients are needed.

## AUTHOR CONTRIBUTIONS

All authors equally accessed the data and contributed to the preparation of the manuscript. IE and MKMS drafted the manuscript. TDH and NOV critically reviewed and edited the manuscript. JPS reviewed the manuscript and provided the genetic counseling.

## FUNDING INFORMATION

Not applicable.

## CONFLICT OF INTEREST

The authors have disclosed that they have no significant relationship with, or financial interest in, any commercial companies pertaining to this article.

## ETHICAL APPROVAL

No ethical approval was needed, as all personal information was anonymized and the identification of our patient is not possible.

## STATEMENT OF HUMAN AND ANIMAL RIGHTS

The present article does not contain any studies with human or animal subjects performed by any of the authors.

## CONSENT

Written informed consent was obtained from the patient to publish this report in accordance with the journal's patient consent policy.

## Data Availability

Data sharing is not applicable to this article as no datasets were generated or analyzed; during the current study.
